# Community-based nursing: a concept analysis with Walker and Avant’s approach

**DOI:** 10.1186/s12909-023-04749-5

**Published:** 2023-10-12

**Authors:** Arezoo Zeydani, Foroozan Atashzadeh-Shoorideh, Meimanat Hosseini, Sima Zohari-Anboohi

**Affiliations:** 1 Student Research Committee, School of Nursing and Midwifery, Shahid Beheshti University of Medical Sciences, Tehran, Iran; 2 Department of Psychiatric Nursing and Management, School of Nursing and Midwifery, Shahid Beheshti University of Medical Sciences, Tehran, Iran; 3 Department of Community Health Nursing, School of Nursing and Midwifery, Shahid Beheshti University of Medical Sciences, Tehran, Iran; 4 Department of Medical Surgical-Nursing, School of Nursing and Midwifery, Shahid Beheshti University of Medical Sciences, Tehran, Iran

**Keywords:** Community-based, Concept analysis, Nursing, Walker and avant

## Abstract

**Background:**

Community-based nursing in recent years has received much attention from nursing schools in different countries as a suitable solution in response to existing and future problems and challenges, but there is yet no comprehensive and correct understanding of this concept and considering its importance, the present study was conducted to the aim of analyzing the concept of community-based nursing.

**Methods:**

Concept analysis was done using Walker and Avant's 8-step approach. Nursing dictionary, Persian dictionary, research articles, journals and conferences articles, dissertations, thesis, books, and other sources related to the concept of research were investigated through search engines and available databases using the keywords of nursing, community-based, concept analysis and Walker and Avant from 1990 to 2023. Finally, 54 articles related to the concept were reviewed and analyzed.

**Results:**

The results showed that community-based nursing has attributes such as individual-oriented/ family-oriented/ community-oriented, social partnership with the communities and stakeholders, social justice, and group and interprofessional cooperation, the community as the main activity setting, providing services based on cultural diversity, providing services according to the context, conditions and community needs, caring for individuals and families with health problems throughout life, responding to the community needs, community-based experiences and facing real-life issues in the context of community, using a problem-based and service-based approach, providing context-based care and considering factors affecting health. In this regard, borderline and related cases (community health nursing, community-oriented nursing, population-based nursing, and public health nursing) were also presented to clarify the concept. Antecedents of community-based nursing included: determining the position of community-based nursing, making infrastructure and structure, the partnership between university, hospital and community, identifying all settings, the presence of educators proficient in education, survey of community needs, having knowledge, communication and community-based skills, expanding the role of the nurse, stakeholders' attitude towards community-oriented nursing and management and financial support. Consequences of community-based nursing included: competence development in nurses, solving community-based nursing challenges, meeting the health needs of individuals, families and communities, social justice, and increasing access to health care services.

**Conclusion:**

The results of this study can provide an objective and understandable image of the use of community-based nurses and their education in practice. Conducting more quantitative and qualitative studies about community-based nursing is also recommended.

**Supplementary Information:**

The online version contains supplementary material available at 10.1186/s12909-023-04749-5.

## Background

The term community-based has different meanings, but the common and main point of all of them is the community, which is the focus of service delivery and where community participation is very important [1]. Changes in health care services have led to changes in nursing. The practice of nursing has changed from providing services in the hospital to the community level [2].

Community-based nursing provides nursing care to individuals, families, and groups wherever they are, such as where they live and work [3]. Community-based nursing in recent years has received much attention and empowers nurses to work at the community level [4–6].

Due to the extensive changes, including the increase in urbanization and the increase in the elderly population, changes in the geographical epidemiology of diseases, and the inability to control them by the health team has been highlighted the need to pay attention to community-based nursing as a suitable solution in response to the problems and challenges ahead. In Iran, a study showed that community-based nursing provides direct access to health and treatment services through home visits and home care and accurate identification of the patients’ needs [7].

Community-based nursing as a new field in education and a new role of nurses in the community has attracted the attention of many nursing schools in different countries in recent years. Still, due to the attention to this concept in recent years, there is no comprehensive and correct understanding. Many people have confused this concept with community-oriented nursing, community health nursing, population-based nursing, and public health nursing, or use these concepts interchangeably. In contrast, these concepts have different meanings and applications, and there is a limited understanding of this concept [3, 8, 9]. As a result, clarifying the concept of community-based nursing and the elements and attributes of this concept increases understanding and the need to pay attention to it.

Also, considering that every community has different context and characteristics, therefore specifying the definition, features, and constituent elements of this concept by examining the concept in other communities helps us in developing knowledge and a comprehensive understanding of the concept [10].

“In fact, without a clear understanding of the concept of community-based nursing, one cannot reasonably anticipate its effective execution and appropriate training. A comprehensive grasp of the concept and its constituent elements is essential for the proper implementation of community-based nursing care, enabling nurses to embody the role of a community-based nurse. Furthermore, such comprehension serves to garner increased attention from policymakers and raise public awareness.“ [2, 3].

In the field of “community-based nursing education” conceptualization has been done by Mtshali [9, 11], but “community-based nursing” has not been conceptually analyzed so far. Therefore, the main aim of this research is to explore the concept of community-based nursing to reach a comprehensive and common understanding of this phenomenon.

## Methods

### Methodological framework

Concept analysis has been widely considered and supported as a fundamental research approach to expanding and developing nursing knowledge. Concept analysis is a process of examining the main elements of the concept that the researcher wants to better understand the concept by reviewing its components. It’s a way to deconstruct a term to understand it better and create a correct definition that provides the possibility of measuring the concept and a great insight into the phenomenon of interest [12]. The purpose of concept analysis is to examine the structure and function of the concept. The concepts within themselves have attributes that make them unique from other concepts. Therefore, concepts are a group of information with defining characteristics. The understanding of the concept changes over time, which is one of the reasons why concept analysis should not be considered a final product. The aim is to understand it in the present moment in time [13]. Walker and Avant’s concept analysis method is a modified and simplified version of Wilson’s (1963) classic concept analysis, which has eight steps instead of 11 steps and is easier for beginner researchers to understand and do it. This logical positivist approach can clarify a concept by simplifying it [14].

### Data sources

This study is a systematic analytical approach; it aims to clarify the concept of community-based nursing and determine its dimensions using the approach of Walker and Avant (2019). To find meanings related to the concept, an extensive search of the literature of 1990–2023 in search engines and available databases such as Iran Doc, Google Scholar, SID, OVID, CINAHL, Scopus, PubMed, Magiran with keywords of Community-based, nursing, Walker & Avant, concept analysis was done.

### Data analysis

In the current research, based on the approach of Walker and Avant (2019), the following steps were carried out, including selecting the concept, determining the aims of the analysis, identifying the uses of the analyzed concept, determining the defined attributes of the concept, identifying a model case, identifying borderline and related issues, identification of the antecedents and consequences of the concept and defining the empirical referents [14].

### Data collection

**Fig. 1:**
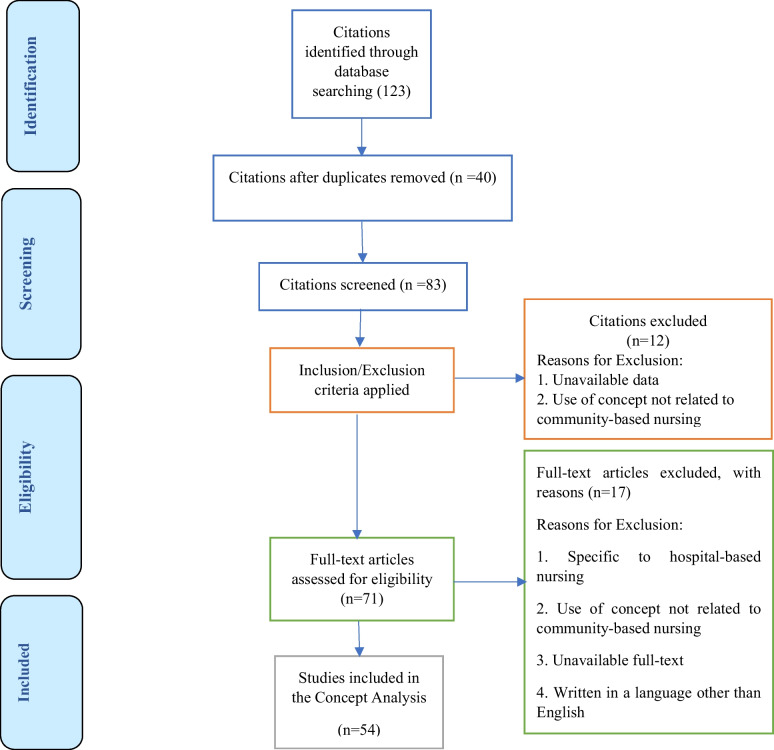
PRISMA diagram of search strategy

In this way, in the beginning, a search was conducted to find what existed under the title of community-based nursing in related articles and sources. Then the articles were included in the study based on the inclusion criteria (English language, relevance to the concept of community-based nursing and similar concepts, access to the full text of the article, and non-repetition). The exclusion criteria included the focus of the study on hospital nursing. The procedure for selecting studies using PRISMA diagrams is shown in Fig. 1. Initially, 123 studies were identified, after removing 40 duplicates, 12 studies based on inclusion and exclusion criteria, 17 studies based on eligibility criteria, finally, 54 studies related to the review concept and defining attributes were extracted from them (Supplementary file).

## Results

### Selection of a concept

In this analysis, the concept of “community-based nursing” is considered the main concept of the present research due to its wide application in response to the challenges and health problems of the community, the ever-increasing changes in the way of providing services and the health care system and its importance in education. This concept has received a lot of attention due to its importance in promoting the health of society in the healthcare system. Still, this concept has no proper understanding and clarity, and the boundaries that separate it from other concepts have not been defined. Therefore, it seems that the clarification of this concept can help to understand the performance of community-based nursing and how to train nurses in this field [10, 15, 16].

### Determine the aims or purposes of analysis

Considering that the concept of community-based nursing is one of the concepts that is confused with many other concepts and is sometimes used instead of other concepts, while these concepts are different from each other [15] and it is necessary to clarify the boundaries and its difference to the other similar concepts, that this issue increases the understanding of this concept and its better application in nursing, the concept analysis of the “community-based nursing” was done.

### Determine the defining attributes

To identify the characteristics of community-based nursing, a wide range of old to new literature (1995–2023) was reviewed, and the characteristics of community-based nursing were determined as follows:

(1) Individual-oriented/family-oriented/ community-oriented, (2) Social partnership with the communities and stakeholders, (3) Social justice, (4) Group and interprofessional cooperation, (5) The community as the main activity setting, (6) Providing services based on cultural diversity, (7) Providing services according to the context, conditions and community needs, (8) Caring for individuals and families with health problems throughout life, (9) Responding to the community needs, (10) Community-based experiences and facing the fundamental issues of life in the context of community, 11. Using a problem-based and service-based approach, 12. Providing context-based care and considering factors affecting health (physical, psychological, political, economic, social, and cultural conditions).

### Identifying all uses of the concept

The concept of co

### Identify antecedents and consequences

lowing cases: (1) Managing acute and chronic conditions and providing acute or chronic care in health care centers, homes, schools, primary care clinics, outpatient services, and community centers [2, 3], (2) Disease prevention and community health promotion [17], (3) Focusing on caring for the illness of individuals and families throughout life and promoting self-care in them [2, 3], (4) Serving in underdeveloped and under-resourced conditions [9], serving at-risk populations (including agricultural workers, industrial workers, pregnant women, people with disabilities, mothers who have recently given birth, etc.), establishing fairness and justice in health, and working to the policymakers to change policy and make the environment healthy [17], (5) Identifying the target population’s needs and meeting the community’s health needs and responding to them, symptoms, and medication management [16, 18].

### Identify a model case

A nurse has gone to their home to take care of a family that has an elderly father with diabetes and a diabetic foot ulcer who needs to change the dressing (attributes including community-oriented, the community is the main activity setting, caring for individuals and families with health problems throughout life). First, the nurse evaluates the condition of the patient’s family in terms of economic, cultural, social, physical and psychological aspects (attributes including providing context-based care and considering factors affecting health) and realizes that their children are all married and they live alone and economically, they are at an average level, as a result, to the patient’s consent, the nurse prepares and uses a suitable dressing for his leg wound (attributes including providing services according to the context, conditions and community needs), and then the nurse teaches the father of the family how to take care of the dressing and the leg wound (individual-oriented) and meanwhile, the nurse asks about his diet, the client does not like some foods and on the contrary eats some foods that are not suitable for him and says that he cannot have a regular meal plan (using a problem-based and service-based approach), also, he does not use some foods at all due to the prohibition in their culture and religion (attribute of cultural diversity). Based on this, the nurse prepares the best meal plan in consultation with the treatment team and nutrition consultant and then implements it with the approval of the specialist doctor and other members of the treatment team, as well as with the client’s consent (attributes including group and interprofessional cooperation and participation). The nurse also teaches his wife about diet and asks her to cooperate with her husband to implement this plan. While talking to the mother of the family, the nurse notices her respiratory distress, asks her questions, examines the mother, and realizes that she has asthma and does not use her respiratory aid sprays properly. As a result, the nurse helps her to use the spray correctly and teaches her. The mother of the family states that she is depressed due to her difficult situation, the nurse talked to the mental health consultant of the care team about this issue, and an appointment was made to examine the mother of the family and help her (attributes including group and interprofessional cooperation and participation). In the end, the nurse explained the risk factors of diabetes and asked them that if their children have these attributes, they must be evaluated and referred to the health care center of their region (details including family-oriented, community-based experiences and facing real problems of life in the context of the community). Finally, the nurse determines the time of the next visit, advises on social services that can help the family, and ensures that the patient and his family have received all the needed care (attributes including social justice and responding to the community needs).

### Identify borderline and related cases

In this study, borderline and related cases of community-based nursing based on the literature review included the following:

1. Community health nursing: In community-based nursing, the nurse may meet an acute need, but the goal is to strengthen the capacity of the individual and family to take care of themselves. The main goal of community health nursing is to maintain the community’s health, and its secondary goal is to promote self-care among individuals and families. It also provides care, especially for high-risk people and those with infectious diseases. Community-based nursing care is family-oriented, even if it is for an individual. Community health nursing combines nursing theory and public health sciences. It assigns the priorities of prevention, protection, and health promotion, and its responsibility goes beyond the client, individual and family. In community-based nursing, the nurse cares for individuals and families who have health problems, while in community health nursing, the nurse works with people who are generally well and have no symptoms. The roles of community-based nurses and community health nurses are both client-centered and service-centered. They include providing care, education, counseling, client advocacy and support, and case management, which are similar. Still, the main difference between the two is in the group-oriented roles. In community health nursing, the nurse has more group roles, such as the community advocate, who knows what the community wants and needs and solves it with the available resources. In community-based nursing, nurses spend most of their time (85%) in case management, patient education, individual and family counseling, and interdisciplinary practice, while in community health nursing, nurses spend most of their time in finding case and patient education, while both emphasize cultural sensitivity [3, 19].
2. Community-oriented nursing: Many attributes of community-based nursing are shared with community-oriented nursing, but they are different. The purpose of community-oriented nursing is to prevent illness and disability, maintain and promote health, focus on health care for individuals, families and groups in the community, provide medical services to improve the quality of life, provide community diagnosis, health monitoring and assessment and school nursing, while the goal of community-based nursing is to manage acute and chronic conditions, focus on caring for the illness of individuals and families throughout life, determining special care in the community where they are located, home care, disease prevention, and health promotion [9].
3. Population-based nursing: Population-based nursing is a systems approach to a problem for a specific population, but in community-based nursing, the target population may be located anywhere. In population-focused nursing evaluation, the target population and the environment in which the population is located should be examined. These assessments focus on epidemiological, environmental, psychological, cultural, spiritual, technological factors and the availability of community support systems [8, 20].
4. Public health nursing: Public health nurses serve poor people instead of working with the whole people, and this is under cover of community health nursing, which occurs in every field. Public health nursing aims to prevent disease and disability and support the community, with a broad focus on community health and investigating the impact of the health status of the community (resources) on the health of individuals, families, and groups [8].

### Identify antecedents and consequences

Antecedents of community-based nursing based on extensive literature review included the following: (1) Determining the position of community-based nursing, its duties and organizational level in the Ministry of Health and achieving the position of the nurse at all levels of health from prevention to rehabilitation [21] and creating job opportunities [7], (2) Making infrastructure and providing structure (political and legal, security, cultural, communications, transportation, facilities, equipment and resources) [7, 16, 18, 22–25], (3) The partnership between university, hospital, community and community health service providers [16, 23, 26], (4) Identifying all areas and capabilities of providing health services and accessing them [27, 28], (5) The presence of educators proficient in community-based nurse education [28, 29], (6) Survey of community needs [29, 30], 6. Survey of community needs [31, 32], (7) Knowledge, communication and community-based skills [33], (8) Expanding the role of the nurse [10, 34, 35], 9.Stakeholders’ attitude towards community-oriented nursing [16, 36], 10. Management and financial support for the provision of community-based nursing services [18, 24, 30, 37].

Based on an extensive literature review, community-based nursing consequences included: 1. Competence development in nurses, such as improving professional, practical, communication skills, critical thinking, teamwork, experience, and deep knowledge about health and social issues in the community [33],

2. Solving community-based nursing challenges such as hospital-oriented and treatment-oriented in the health system, defects in the position and role of community-based nurses, flaws in community-based education infrastructure, deficiencies in trust, awareness, and acceptance of nurses in the community by the people [7, 38, 39], 3. Meeting the health needs of individuals, families, groups, communities and populations, developing community capacity for health, social justice, and eliminating health inequality [11, 12, 16, 23, 30, 40–44], 4. Increasing access to health care services [7, 11].

### Define empirical referents

According to the extensive literature review, community-based nursing is a vital approach that delivers essential care across the lifespan with a central focus on enhancing overall health, primary care, and rehabilitation. This approach thrives on interdisciplinary collaboration to cater to diverse client groups within their natural environments. It is firmly rooted in the principle that healthcare decision-making primarily rests with the individual, their family, and the community. The nurse plays a pivotal role in devising nursing interventions for the client, their family, and the healthcare team, aligning these interventions with the values held by the client, their family, and the broader community. Community-based nursing places a strong emphasis on prevention, striving to avert the onset of diseases, promptly identify health issues, and provide early intervention and rehabilitation following illness or injury [3].

## Discussion

In the present study, the concept of community-based nursing was analyzed using Walker and Avant’s approach. Since the concept analysis causes objectification of a specific concept and its operationalization [14], it seems that expressing experimental interpretations of community-based nursing can be applied more quickly in the hospital, community, and education. Many studies provide positive evidence of community-based nursing practice [2, 17, 24, 33, 40]. As it was mentioned, based on a review of studies, community-based nursing has several attributes that are mentioned below.

Community-based nursing is characterized by individual-centered, family-centered, and community-centered orientation. It provides nursing care for individuals, families, and groups wherever they are, including their place of residence, workplace, school, etc. Many studies have considered this attribute important in community-based nursing [3, 16, 18]. In such a way, even if the individual is a client of the community-based nurse, nursing care should be family-oriented and consider the needs of the individual and the family. Being family-oriented means that the nurse believes in improving families’ competence and designs care based on the family’s needs and decisions; this increases the independence of the individual, family and their participation. Such care is necessary for the community because most clients live in their homes despite their health problems [3].

Social participation is defined as a person’s participation in activities that interact with others in community, also, this concept is defined as groups that work with common goals, responsibilities and power for the betterment of community and it includes the participation of community members, governmental and non-governmental organizations, universities, health center staff and other stakeholders and pays attention to the populations it is supposed to serve, many studies have mentioned social participation as an essential characteristic of community-based nursing [3, 9, 11, 12, 16, 18, 22–24, 29, 37, 40, 41, 45–49] and the success of community-based nursing performance depends to a large extent on this factor, because it will not be possible to achieve the goals without involving individuals, families and community, also, providing care by a community-based nurse should be focused on the values, preferences of the individual and the family, Therefore, it is necessary to involve them in order to succeed in reaching the goals and supporting the individual and the family [18]. Community participation provides an opportunity for all community members to participate actively and effectively in the process of development and exploitation, and health promotion programs seek the participation of the community and stakeholders as active partners. Hence the category of participation is considered one of the crucial characteristics of community-based nursing [50].

The meaning of social justice is the fair and equitable benefit of the people of a community from health care based on need, which is at the heart of community-based health promotion measures to achieve health equality and is another essential attribute of community-based nursing [51].

 Community-based nursing requires group and interprofessional cooperation, and the nurse collaborates with different teams, including doctors, pharmacists, specialists, and assistants. Interdisciplinary collaboration is an essential element in the role of community-based nurses because nurses cannot achieve patient support goals without collaboration with other healthcare team members. Nurses provide the necessary care throughout the patient’s life, focusing on improving health and primary rehabilitation care through interdisciplinary cooperation for different community Sects. [2, 10]. In fact, in community-based nursing, to provide comprehensive support and integrated services, it is necessary for medical, administrative, human services, and related professionals to cooperate. It requires extensive cooperation of the government organization with other related institutions such as the welfare organization, municipalities, radio, and television. Many studies have emphasized collaboration as an essential attribute of community-based nursing [10, 12, 16, 18, 22, 24, 29, 37, 40, 45, 49, 52].

The community is considered the main setting for the activity of community-based nurses. It is used to such an extent that the percentage of community-based experiences is higher than other clinical experiences. Most studies have emphasized this issue [8, 9, 11, 16, 23]. Another issue is that the hospital is considered a part of the community. Still, nurses perform most of their activities at the community level and provide services according to the prevention levels of healthy people in their natural living environments, from hospital to rehabilitation [16].

Another essential attribute of community-based nursing is attention to cultural diversity. Cultural diversity exists within and between countries, and nurses are morally committed to providing care appropriate to the culture. To provide adequate care to a client with a different culture or ethnic background, the nurse tries to understand the other person’s point of view regarding their cultural framework. When the nurses are not successful in this field, the consequence will be inequality in care; considering ethnic diversity in Iran, understanding the beliefs of clients, patients, and families, and paying attention to their needs deepens the relationship between nurses and clients [53]. The nurse must be aware of cultural differences, value the patient’s culture, include it in care plans, and communicate one-by-one with people and families with diverse ethnic or cultural backgrounds in such a way that shows respect for their culture. This brings mutual satisfaction between nurse and client [3, 17]. The emphasis is that community is inextricably linked with cultural values. Many studies have considered cultural diversity as one of the essential pillars of community-based nursing [16–18, 29].

Providing nursing care considering the background, conditions, and community needs is one of the attributes of community-based nursing since the community is the primary activity setting, taking into account the local, regional, and global community conditions, demographic and epidemiological developments, the prevalence of mental diseases, severe changes and the burden of diseases and developing a care plan based on that importance has many and different studies have emphasized this issue [26, 34, 41, 49].

The philosophy of community-based nursing is to guide nursing care for individuals, families with health problems, and other groups throughout life, wherever they are, including where they live, work and go to school, etc. According to this definition, community-based nursing is not a specialty but a philosophy that guides all nursing care [3] and have been mentioned in different studies [3, 54].

Community-based nurses are responsible for the health needs of the community. They must be able to provide the necessary care for individuals and families and investigate, plan, intervene, and evaluate the community’s needs. Since nursing services are mainly offered in hospitals in Iran, the activities of nurses do not meet the community’s needs, and it is one of the essential things mentioned in many studies [11, 16, 18, 34, 45, 49, 55, 56]. The results of Baqhaei et al.‘s study also showed that the need to train capable and competent nurses who respond to the changing needs of the community has increased, for example, with the increase of the elderly population, palliative care, prevention, and acute care are more important [57].

Community-based experiences and facing real-life problems in the context of the community are integral parts of community-based nursing. Nurses need a wide range of experiences to provide care to individuals, families, and communities, from preventive care to acute care and rehabilitation. This means that they need direct access to population groups to work with and communicate with over time and help improve their health status, and many studies have emphasized this issue [16, 22, 23, 37, 40, 42, 58].

Using a problem-based and service-based approach to solve problems is one of the attributes of community-based nursing. Solving problems and dealing with real-life issues requires using such an approach, and by focusing on the situation, nurses look for different solutions. With critical thinking and using evidence, they provide the best available resolution to meet the needs of the client and the family [11, 16, 49] and serve the community, especially under-resourced communities. Different studies have considered and emphasized the importance of these approaches in community-based nursing [11, 16, 49, 55].

Due to being exposed to the conditions and realities of the lives of individuals and families, nurses need to be aware of the target community’s values. This issue makes them aware of social and cultural issues, injustices, and other factors affecting health [9].

Proper care of individuals and families in social environments requires careful attention to social risks such as poverty, mental illness, unsafe housing, history or current injury, malnutrition, transportation problems, low literacy, etc. The nursing care team should comprehensively assess these areas and cooperate with social partners and colleagues (such as welfare, nursing home, etc.) to deal with them and follow the needs of the individual, family, and community over time. In community-based nursing, nurses are exposed to social, economic, political, cultural and other factors affecting the health of individuals, families, and communities. It is believed that such exposure facilitates a better understanding of social issues and equips nurses with the skills to deal with them. More importantly, it provides a comprehensive and complete view of health and disease because when the nurse encounters the patient only in the hospital setting, such opportunities are lost [2, 3, 9, 16, 18, 30, 37].

To ensure the continued provision of essential services, community-based nurses must hold a well-defined position within the healthcare infrastructure [22, 39, 59]. Their roles and responsibilities should be clearly delineated, and the settings for their activities must be precisely defined. Equally important is the need for these nurses to acquire the necessary skills through expert-led training programs, thus enabling them to deliver effective services [39, 45]. It is imperative that policymakers adopt a community-oriented perspective, as their support is pivotal for the realization of these goals [36].

Being in different situations and solving problems in community increases the competence of nurses [33]. The positive result of the presence of nurses in the community creates trust in the community and by increasing access to health and treatment services, the needs of the community are met. Increasing access to health for all helps to eliminate health inequalities [10, 12, 26]. As a result, more people turn to this type of service and visits to hospitals decrease. Such a thing reduces many costs and as a result, policy makers pay attention to the needs of the community and they try to solve the problems and challenges in this field [44, 60].

## Limitation

Concept analysis of community-based nursing focused only on theoretical analysis without empirical verification from the nurse educators; this indicates the limitation of the study. Verification of the concept from the nurse educators could have helped the researcher obtain additional data to expand further or clarify the concept.

## Conclusion

Proper care of individuals and families in social environments requires careful attention to social risks such as poverty, mental illness, unsafe housing, history or current injury, malnutrition, transportation problems, low literacy, etc. The nursing care team should comprehensively assess these areas and cooperate with social partners and colleagues (such as welfare, nursing home, etc.) to deal with them and follow the needs of the individual, family, and community over time. In community-based nursing, nurses are exposed to social, economic, political, cultural and other factors affecting the health of individuals, families, and communities. It is believed that such exposure facilitates a better understanding of social issues and equips nurses with the skills to deal with them. More importantly, it provides a comprehensive and complete view of health and disease because when the nurse encounters the patient only in the hospital setting, such opportunities are lost. Based on the literature review, it can be said that community-based nursing, in facing the fundamental problems of life, using a problem-oriented and service-oriented approach, provides the necessary health care for individuals and families to the health problems during life and based on the context and community needs (cultural, political, social, economic, health status of the client) provides from the first level of the prevention to the third level and it does this through group and interdisciplinary cooperation and taking into account cultural diversity, factors affecting health and social justice for different strata of community in the natural environment of life and places in the community and it is based on the principle that community-based nursing is a collaborative work, the individual, family, and community have primary responsibility for health care decisions and the nurse mainly determines the nursing interventions with the client, the family and the health care team based on the values of the client, the family and the community and tries to respond to the community needs. The main goal of community-based nursing is to strengthen the capacity of the individual and family to take care of themselves and improve the community’s health. To achieve such a goal, it is necessary to provide infrastructure and structures such as the position of a community-based nurse, essential resources, and facilities and conditions for the nurse to enter the community. In line with the present study, it is proposed to investigate the challenges of community-based nursing education in Iran and introduce a solution to improve it.

### Supplementary Information

**Additional file 1: Supplementary file.** Overview of all included studies in concept analysis

## Data Availability

All data generated or analyzed during this study have been incorporated into this manuscript.
