# Supplementary material for: Community-based nursing: a concept analysis with Walker and Avant’s approach
Source: BMC Med Educ. 2023 Oct 12;23:762. doi: 10.1186/s12909-023-04749-5 (PMC10571235; doi:10.1186/s12909-023-04749-5)
Supplement: Supplementary file 1 — Additional file 1: Supplementary file. Overview of all included studies in concept analysis [file 12909_2023_4749_MOESM1_ESM.docx]

| Supplementary file: Overview of all included studies in concept analysis | | | | | | |
| --- | --- | --- | --- | --- | --- | --- |
| N | Author, year | Title | Aim | Methods | Setting | Key findings |
| 1 | Whelan, 1995  (1) | The health corner: a community-based nursing model to maximize access to primary care | This paper describes a primary health care delivery that addresses the specific health concerns of a neighborhood. | Descriptive | Community center | Community collaborative and community's active participation  Identifying the health needs of the neighborhood in the community  Responding to the needs of community  Reducing medical care costs  Increasing access to care services  Improving the health status of the community  Collaboration |
| 2 | Baldwin, 1998  (2) | Population‐focused and community‐based nursing—moving toward clarification of concepts | The purpose of this paper is to share the authors' thinking about what it is that makes population-focused nursing “different” from community-based practice | Examination of old and new terms and concepts | Community | Similar but different concepts (Community-based nursing, community-oriented nursing, community health nursing, population-based nursing, and public health nursing).  The community is the main setting for the activity of community-based nurses.  Percentage of community-based experiences is higher than other clinical experiences.  Avoid cost increases  Evaluation of epidemiological factors  Working with groups |
| 3 | Flynn, 1998  (3) | Communicating with the public: community-based nursing research and practice | This paper presents examples of community-based nursing research in relation to selected health care trends that affect practice and considers ways the media can help community health nurses be more effective communicators with the public. | Descriptive | Community | Increasing the effectiveness of services through media advocacy  Disease prevention and community health promotion  Serving at-risk populations  Establishing fairness and justice in health  Working to the policymakers to change policy and make the environment healthy  Cost control  Focus on disease prevention and health promotion  Serving people across the lifespan and culturally diverse populations  Developing community capacity for health |
| 4 | Beauchesne, 1999  (4) | An interdisciplinary community-based educational model | The purpose of this article is to describe an innovative partnership among academia, community, and service to better meet the health care needs of underserved populations. | Descriptive | Community-based settings and health centers | Social participation is an essential characteristic of community-based nursing  The collaboration and participation with other organizations and professions.  Responding to the health needs of the community  Increasing access to health care with community-based nurse services  Training of community-based nurses by experts  Advocate for the client  Cultural sensitivity  Team working |
| 5 | Siegrist, 1999  (5) | Concerns of baccalaureate nurse educators in Kentucky regarding community-based nursing education | -  Book | - | Community | Community health nursing is different from community-based nursing  Providing care to individuals and families wherever they live  Management of chronic and acute conditions throughout life  Helping clients adapt to health problems |
| 6 | Whitehead, 2000  (6) | The role of community-based nurses in health promotion | This account offers a viewpoint on how the profession could move forward on this complex issue. | Descriptive | Community | Expanding the role of the nurse  Community nursing ability to fulfil its health-promoting role  Extensive cooperation  Providing the necessary care throughout the patient's life with a focus on improving health and primary rehabilitation care through interdisciplinary collaboration for different parts of community.  Health promotion  The importance of community-based nurses |
| 7 | Fichardt, 2000  (7) | Adapting to and implementing a problem-and community-based approach to nursing education | The aim of this article is to describe how, with the passage of time, the staff of the School of Nursing, rather than merely concentrating on change, regarded change as a process of successive challenges. | Descriptive | Community | Providing nursing care considering the background, conditions, and community needs  Social participation  Problem and community-based approach  Considering the needs of communities  Cultural competence |
| 8 | Villani, 2000  (8) | Community-based education | About the community-based education | Descriptive | Community | Connect with the community  Community involvement  Emphasis on community  Recognizing and supporting the needs of community |
| 9 | Oros, 2001  (9) | Community-based nursing centers: challenges and opportunities in implementation and sustainability | Presenting the challenges and opportunities of community-based nursing | Descriptive | Community | Community-based nurses are responsible for the health needs of the community.  Providing the necessary care for individuals and families  Community participation  Access to health care  Community based experiences |
| 10 | Edwards, 2002  (10) | Transition to community-based nursing curriculum: processes and outcomes | This article describes one college's process of curricular change from a traditional to community-based format. | Qualitative descriptive study | Community setting | Partnership with the communities  Community involvement  Meeting the health needs  Developing community capacity for health  Positive practice of community-based nurses  Social participation  Interdisciplinary collaboration  Direct access to the community  The need for community-based experiences  Providing chronic and acute care in community settings |
| 11 | De Villiers, 2004  (11) | Evaluation of clinical teaching and professional development in a problem and community-based nursing module | The purpose of the study was to evaluate a first-year clinical teaching module as part of an extensive programme. | Quantitative research method (a descriptive design) | Community | Social participation  Collaboration  Providing nursing care considering the background, conditions, and community needs  Responding to the needs of community  Using a problem-based and service-based approach to solve problems  Team working  communication skill |
| 12 | Uys, 2004  (12) | Curriculum development in nursing: Process and innovation | -  Book | - | Community | Identifying the target population's needs and meeting the community's health needs and responding to them, symptoms, and medication management  The partnership between university, hospital, community and community health service providers  Expanding the role of the nurse  Stakeholders' attitude towards community-oriented nursing  Meeting the health needs of individuals, families, groups, communities and populations, developing community capacity for health, social justice, and eliminating health inequality  Providing care where individuals, families and communities live  Social participation  Cooperation and collaboration  Multidisciplinary team approach  The community is the main setting of the activity  A higher percentage of community-based experiences than other clinical experiences  Providing services from prevention to rehabilitation in the community  Considering cultural diversity  Using a problem-based and service-based approach to solve problems  Access to groups  Attention to the factors affecting health and social issues  communication skill  Considering cultural diversity |
| 13 | Mtshali, 2005  (13) | Conceptualisation of community-based basic nursing education in South Africa: a grounded theory analysis | The purpose of the study was to analyse the concept ‘community-based education’ with the aim of discovering shared understanding of this phenomenon in basic nursing education within the South African context. | Strauss and Corbin’s (1990) grounded theory approach | Community | Different concepts (community-oriented nursing, community health nursing, population-based nursing, and public health nursing)  Serving in underdeveloped and under-resourced conditions  Social participation  The community is the main setting of the activity  A higher percentage of community-based experiences than other clinical experiences  Awareness of social and cultural issues, injustices and other factors affecting health  Proper care of individuals and families in social environments  Attention to social risks  Responding to the needs of community |
| 14 | Feenstra, 2006  (14) | Managing community and neighborhood partnerships in a community-based nursing curriculum | Explanation of the role and performance of the community partnership coordinator and neighborhood coordinator | Descriptive | Community | Management and financial support for the provision of community-based nursing services  Positive practice of community-based nurses  Social participation  Cooperation and collaboration  Examining the needs of the community  Infrastructure development for community-based nursing practice |
| 15 | Mtshali, 2009  (15) | Implementing community-based education in basic nursing education programs in South Africa | The purpose of this study was to analyse the implementation of community-based education in basic nursing education programmes in South Africa. | Strauss and Corbin's (1990) grounded theory approach | Community | The partnership between university, hospital, community and community health service providers  Providing nursing care considering the background, conditions, and community needs  Government support  The community is the main setting of the activity  The partnership between university, hospital, community and community health service providers  Providing nursing care considering the background, conditions, and community needs  Government support  The community is the main setting of the activity  Reducing inequality in access to health services |
| 16 | Wee, 2010  (16) | The pedagogical value of a student-run community-based experiential learning project: the Yong Loo Lin School of Medicine Public Health Screening | Assessing the pedagogical value of a student-led community-based experiential learning project | Cross sectional | Community | Communication skill  Team working  Identifying social issues |
| 17 | Kaye, 2011  (17) | Lessons learnt from comprehensive evaluation of community-based education in Uganda: a proposal for an ideal model community-based education for health professional training institutions | The objective was to make a comprehensive assessment of CBE as implemented by Ugandan health professional training institutions to document the nature of CBE conducted and propose an ideal model with minimum requirements for health professional training institutions in Uganda. | Qualitative | Community | Social participation  Need financial support |
| 18 | Mwanika, 2011  (18) | Perception and valuations of community-based education and service by alumni at Makerere University College of Health Sciences | This study surveyed the alumni of Community-Based Education and Service (COBE) at Makerere to obtain their perceptions of the management and administration of COBE and whether COBE had helped develop their confidence as health workers, competence in primary health care and willingness and ability to work in rural communities. | Mixed qualitative and quantitative study | Community | Making infrastructure and providing structure (political and legal, security, cultural, communications, transportation, facilities, equipment and resources)  Communication skill  Team working  The positive effect of community-based nurse performance  Ensuring fair access to people  Financial support  Structural support |
| 19 | Ladhani, 2012  (19) | Competencies for undergraduate community-based education for the health professions – A systematic review | The systematic review aimed at identifying and categorizing CBE competencies implemented in nursing and medical schools to inform all stakeholders of health professional's education. | Systematic review | Community | Cultural competence  Considering the needs of community  Community participation  Communication skill |
| 20 | Hamad, 2012  (20) | Community based education: what? why? how | Define the meanings of community-based and community-oriented medical education | -  Book | Community | Responding to priority health needs of the community  The impact of psychosocial, economic, cultural and political factors on the health of individuals, families and communities  Community participation |
| 21 | Linda, 2013  (21) | Lived experiences of a community regarding its involvement in a university community-based education programme | This study investigated the experiences of a community regarding its involvement in a community-based education programme offered by a university nursing school in Durban, South Africa. | Qualitative (interpretive existentialist-phenomenological design) | Community | Community involvement and participation |
| 22 | Nowak, 2015  (22) | Community partnerships: teaching volunteerism, emergency preparedness and awarding red cross certificates in nursing school curricula. | It reviews teaching strategies that were evaluated using an experimental design examining disaster preparedness skill formation and partnerships with volunteer disaster relief agencies. | Quasi experimental pre-test and post-test two groups (control group) | Community | Social participation  Working in an interdisciplinary team |
| 23 | Kim, 2015  (23) | The effect of a community-based self-help intervention: Korean Americans with type 2 diabetes | The purpose of this study was to test the effectiveness of a community-based, culturally tailored, multimodal behavioral intervention program in an ethnic/linguistic minority group with type 2 diabetes. | RCT  (Intervention and control group) | Naturally occurring community setting | Considering culture in providing care to people with diabetes  Reductions in hemoglobin A1c in intervention group in a community-based program  Statistically significant improvement in diabetes-related self-efficacy and quality of life when compared with the control group |
| 24 | Stricklin, 2016  (24) | Achieving clinical competencies through community-based clinical experiences | This study aims to investigate how prelicensure undergraduate baccalaureate (BSN) student nurses and their faculty perceive students’ ability to achieve learning outcomes in community-based PMH clinical settings. | Qualitative descriptive study | Community-based clinical settings | Community-based experiences  Facing real-life problems in the context of the community  Enhancing competence through community-based experiences  Direct access to population groups to work with and communicate with over time and help improve their health status  Management of acute and chronic conditions |
| 25 | Amalba, 2016  (25) | The perceived usefulness of community-based education and service (COBES) regarding students' rural workplace choices | The purpose of this study was to investigate students’ perceived usefulness of COBES and its potential effect on their choice of career specialty and willingness to work in rural areas. | Mixed method cross sectional study design | Community | Community partnerships  Meeting the health needs  Social justice  The community is considered the main setting for the activity  Community-based experiences  Improvement of structure such as improvement of transport arrangements, accommodation, equipped facilities |
| 26 | Mtshali, 2016  (26) | Community-based nursing education in South Africa: A grounded-middle range theory | The purpose of this study was (a) to conceptualize the phenomenon CBE within the South African context, and (b) to develop a middle-range theory that would guide the practice of CBE in basic nursing education in the country. | Grounded theory approach | Community | Meeting the health needs.  Increasing access to health care services  Social participation.  The community is the main setting for the activity of community-based nurses.  Greater percentage of community-based experiences than clinical experiences.  Being responsive to the needs of community.  Using a problem-based and service-based approach to solve problems. |
| 27 | Lubbers, 2016  (27) | The effects of pediatric community simulation experience on the self-confidence and satisfaction of baccalaureate nursing students: A quasi-experimental study | The purpose of this study was to determine the effects of a pediatric community simulation experience on the self-confidence of nursing students. | Quasi experimental Pre-test and post-test Single group | Community | Increasing nurses' self-confidence and the positive impact of community-based activities |
| 28 | Lubbers, 2017  (28) | Satisfaction and self-confidence with nursing clinical simulation: Novice learners, medium-fidelity, and community settings | The purpose of this study was to evaluate the use of medium fidelity simulation by measuring self-confidence and satisfaction among novice learners and to demonstrate the feasibility of using community-based scenarios in simulation. | Quasi experimental Pre-test and post-test Single group | Community | Increasing nurses' satisfaction and the positive impact of community-based activities |
| 29 | Heydari, 2017  (29) | Exploring the position of community-based nursing in Iran: A qualitative study | The aim of this study was to explore the status of community-based nursing in Iran. | Qualitative | Faculties of Nursing | Increasing access to health care  Creating job opportunities  Making infrastructure and providing structure (political and legal, security, cultural, communications, transportation, facilities, equipment and resources)  Increasing access to health care services  Solving community-based nursing challenges (hospital-oriented and treatment-oriented in the health system, defects in the position and role of community-based nurses, flaws in community-based education infrastructure, deficiencies in trust, awareness, and acceptance of nurses in the community by the people) |
| 30 | Asakawa, 2017  (30) | Establishing community-based integrated care for elderly patients through interprofessional teamwork: a qualitative analysis | The goal of this study was to clarify whether the process of developing interprofessional cooperation by studying what those involved in the medical profession feel is necessary to effectively build interdisciplinary cooperation when providing community- based integrated care. | Qualitative descriptive study | Community-based integrated care center and municipalities | Providing comprehensive and integrated services with the cooperation and participation of other organizations and disciplines |
| 31 | Massimi, 2017  (31) | Are community-based nurse-led self-management support interventions effective in chronic patients? Results of a systematic review and meta-analysis | The aim of this systematic review and meta-analysis is to assess the efficacy of the nurse-led self-management support versus usual care evaluating patient outcomes in chronic care community programs | Systematic review | Community setting | Meeting the health needs of individuals, families, groups, communities and populations  Developing community capacity for health  Reducing hospital-based outpatient care costs  Providing community-based services to meet emerging needs |
| 32 | Cohn, 2017  (32) | Community-based models of care delivery for people with serious illness | The study first describes guiding principles that are inherent to the ideal community-based model program and next discuss core competencies that these programs must possess to provide high-quality care. | Descriptive | Community | Identifying the target population's needs and meeting the community's health needs and responding to them, symptoms, and medication management  Making infrastructure and providing structure (political and legal, security, cultural, communications, transportation, facilities, equipment and resources)  Management and financial support for the provision of community-based nursing services  Individual-centered, family-centered, and community-centered orientation  Providing direct care to individuals, families and groups where they live and work  Social participation  Cooperation and collaboration  Responding to the needs of community  considering the cultural diversity  Attention to the social factors that determine health |
| 33 | Parry, 2018  (33) | Assessing levels of student nurse learning in community based health placement with vulnerable families: Knowledge development for future clinical practice | The research project explored the use of a community placement to assist nursing students in applying psychosocial theories to practice; determining the affect of nursing students to the community service; and if the learning achieved in the placement was applicable to their future nursing practice were also objectives of the research. | Mixed method | Community | Understanding factors affecting health  Working with vulnerable populations  Improve accessibility  Participation  Considering the needs of society |
| 34 | Huang, 2018  (34) | Delivery of public health services by community health workers (CHWs) in primary health care settings in China: a systematic review (1996–2016) | The systematic review aimed to identify the types of public health services provided by CHWs and summarized potential barriers and facilitating factors in the delivery of these services. | Systematic review | Community | Identifying all areas and capabilities of providing health services and accessing them |
| 35 | Harvey, 2019  (35) | Developing a community-based nursing and midwifery career pathway – A narrative systematic review | Therefore, the aim of this review is to: 1. define community nursing and midwifery within the context of contemporary specialist practice in Australia. 2. Formulate for the Queensland government both an early career transition pathway for community nursing and midwifery as the first phase of a three-phase study. | Systematic review | Community | The importance of community-based nursing and its performance  Similar but different concepts (Community-based nursing, community-oriented nursing, community health nursing, population-based nursing, and public health nursing). |
| 36 | Walker, 2019  (36) | Strategies for theory construction in nursing | -  Book | - | - | The approach of Walker and Avant (2019): including selecting the concept, determining the aims of the analysis, identifying the uses of the analyzed concept, determining the defined attributes of the concept, identifying a model case, identifying borderline and related issues, identification of the antecedents and consequences of the concept and defining the empirical referents |
| 37 | Kamau, 2019  (37) | Effect of community based health education on knowledge and attitude towards iron and folic acid supplementation among pregnant women in Kiambu County, Kenya: A quasi experimental study | To determine effect of community-based Iron and Folic Acid Supplementation (IFAS) health education, utilizing CHVs, on IFAS knowledge, levels of counselling on various IFAS topics and attitude towards IFAS among pregnant women in Kiambu County. | Pretest-Posttest Quasi-Experimental study design | Community | The success of community-based interventions with community participation and ownership  Facilitating client access  Consider culture |
| 38 | Claramita, 2019  (38) | Community-based educational design for undergraduate medical education: a grounded theory study | The aim to develop a CBE framework for undergraduate medical education to engage students and teachers with better, more meaningful learning, within primary health care settings. | Grounded theory | Community | Community-based nurses should be trained by competent educators.  Survey of community needs  Management and financial support for the provision of community-based nursing services  Meeting the health needs  Collaboration  Understanding the factors affecting health problems |
| 39 | Steffy, 2019  (39) | Community health learning experiences that influence RN to BSN students interests in community/public health nursing | This research explored the community health learning experiences that influenced postlicense nursing students’ interests in a professional role change. | Qualitative | Community-based setting | Community-based experiences and facing real-life problems in the context of the community  Access to demographic groups |
| 40 | Higgins, 2020  (40) | Improving oral health: integrating oral health content in advanced practice registered nurse education | This article describes the development, implementation, and evaluation of a community-based, interprofessional education experience designed to improve oral health and collaborative practice knowledge, skills, and attitudes of advanced practice registered nurse students. | Quasi experimental pre-test and post-test single group | Community-based interprofessional oral health unit | Interprofessional performance  Team working  Access to oral health care  Improving skills in community-based practice |
| 41 | Lestari, 2020  (41) | Stimulating students' interprofessional teamwork skills through community-based education: a mixed methods evaluation | This study aims to evaluate a community-based interprofessional education (CBIPE) programme by exploring the students’ perception toward CBIPE design and toward groups’ teamwork. | Mixed method | Community | Interprofessional performance  Collaboration and team working  Communication and problem solving skills  Access to oral health care  Improving skills in community-based practice |
| 42 | Cheng, 2020  (42) | Experiential learning program to strengthen self-reflection and critical thinking in freshmen nursing students during COVID-19: A quasi-experimental study | This article focuses on the unique needs and concerns of nursing educators and nursing students in the face of the COVID-19 pandemic. | Quasi-experimental study  (One-group, pre-test and post-test design) | A community of real intelligent baby care, maternity experience, and elderly experience | Active participation  Communication and teamwork skills  Community based experience |
| 43 | Aroogh, 2020  (43) | Social participation of older adults: A concept analysis | This study aims to clarify and reduce the ambiguities in the concept of social participation in order to achieve a clear and understandable definition among the elderly people | Concept analysis, Walker and Avant’s 8-Step method | Society | Social participation |
| 44 | Shin, 2020  (44) | Concept analysis of community health outreach | The study aimed to clarify the general definition of community health outreach to facilitate its understanding and use. | Walker and Avant’s (2010) method | Community | Meeting the health needs of individuals, families, groups, communities and populations  Developing community capacity for health  Social justice, and eliminating health inequality  Social participation  Extensive cooperation and collaboration |
| 45 | Ohta, 2021  (45) | The contribution of citizens to community-based medical education in Japan: A systematic review | This research synthesizes the impact of the involvement of communities on the learning of medical trainees in community-based medical education. | Systematic review | Community | Management and financial support for the provision of community-based nursing services  Social participation  Cooperation and collaboration  Community-based experiences and facing real-life problems in the context of the community  Understanding social issues |
| 46 | Barasteh, 2021  (46) | Future challenges of nursing in health system of Iran | This study aims to explore the future challenges of nursing in the health system of Iran from the perspective of nursing experts. | Qualitative | Deputy of Nursing, Nursing Board, Iranian Nursing Organization, scientific and Professional Associations, and nursing schools | Expanding the role of the nurse  Providing nursing care considering the background, conditions, and community needs  Responding to the needs of community |
| 47 | Shirvani, 2021  (47) | Community-based educational interventions for prevention of type II diabetes: a global systematic review and meta-analysis | The objective was to estimate the change in community-based education interventions throughout the world that may effectuate in risk parameters of type II diabetes (T2D), including the diabetes incidence rate, fasting blood glucose, hemoglobin A1C, body mass index, waist circumference, and systolic and diastolic blood pressure. | Comprehensive search and meta-analysis | Community | Providing community-based nursing care for individuals, families with health problems, and other groups across the lifespan  Reducing the incidence of diabetes by community-based nurse interventions |
| 48 | Kerrigan, 2021  (48) | A community-based program to reduce acute rheumatic fever and rheumatic heart disease in northern Australia | The aim is to determine how the model was experienced by study participants. | Qualitative | Communities | Interaction and close communication between the government and health service providers with communities  Meeting the health needs  Social justice, and eliminating health inequality |
| 49 | Hall, 2021  (49) | Social responsibility and community-based research in higher education institutions | -  Book | - | Community | Social participation  Public engagement  Community-university engagement |
| 50 | Nagata, 2021  (50) | Evaluation of a training program for community-based end-of-life care of older people toward aging in place: A mixed methods study | To evaluate a training program that supports community-based service staff in implementing aging-in-place and end-of-life care | Mixed methods | Community | Confidence gains and changes in attitudes by community-based practice  Stakeholders' attitude towards community-oriented nursing |
| 51 | Fauziddin, 2022  (51) | Community-based education and regional culture, has it been put into practice? | This study aimed to obtain the appropriate strategy for implementing community-based education and local culture. | Literature review approach | Community | The presence of educators proficient in community-based nurse education  Survey of community needs  Social participation  Cooperation and collaboration  Considering cultural diversity |
| 52 | Nuuyoma, 2022  (52) | Nursing students' experiences of community-based learning in an undergraduate programme at a Namibian University | The study explored nursing students’ experiences of CBL in an undergraduate programme at a Namibian university. | Qualitative descriptive design | Community | Making infrastructure and providing structure (political and legal, security, cultural, communications, transportation, facilities, equipment and resources)  Social participation  Cooperation and collaboration  Community-based experiences  Providing care to individuals, families, and communities, from preventive care to acute care and rehabilitation  direct access to population groups |
| 53 | Ng, 2022  (53) | A look back: assessment of the learning outcomes of the community-based research experiences of the senior high school students of a higher education institution in Batangas | This study aimed to assess the learning outcomes of the community-based research (CBR) experiences among senior high school students of this HEI in their Practical Research II subject. | Descriptive method | Community | Social participation |
| 54 | Atashzadeh-Shoorideh, 2023  (54) | Explaining of existing challenges of community-based undergraduate nursing education in Iran: a qualitative study | The study was conducted to explain the existing challenges of community-based undergraduate nursing education in Iran. | Qualitative study | Community | The importance of the position and role of the community-based nurse  The importance of infrastructure and structures  Cooperation and teamwork  Gaining people's trust and raising their awareness  The importance of the health paradigm |

1. Whelan E-M. The health corner: a community-based nursing model to maximize access to primary care. Journal of Public Health Reports. 1995;110(2):184.

2. Baldwin JH, O'Neill Conger C, Abegglen JC, Hill EM. Population‐focused and community‐based nursing—moving toward clarification of concepts. Public Health Nursing. 1998;15(1):12-8.

3. Flynn BC. Communicating with the public: community‐based nursing research and practice. Public Health Nursing. 1998;15(3):165-70.

4. Beauchesne MA, Meservey PM. An interdisciplinary community-based educational model. Journal of Professional Nursing. 1999;15(1):38-43.

5. Siegrist BEC. Concerns of baccalaureate nurse educators in Kentucky regarding community-based nursing education: University of Louisville; 1999.

6. Whitehead D. The role of community-based nurses in health promotion. British Journal of Community Nursing. 2000;5(12):604-9.

7. Fichardt A, Viljoen M, Botma Y, Du Rand P. Adapting to and implementing a problem-and community-based approach to nursing education. Curationis. 2000:86-92.

8. Villani CJ, Atkins D. Community-based education. School Community Journal. 2000;10(1):39-44.

9. Oros M, Johantgen M, Antol S, Heller BR, Ravella P. Community-based nursing centers: challenges and opportunities in implementation and sustainability. Policy, Politics, Nursing Practice. 2001;2(4):277-87.

10. Edwards JB, Alley NM. Transition to community-based nursing curriculum: processes and outcomes. Journal of Professional Nursing. 2002;18(2):78-84.

11. De Villiers J, Joubert A, Bester C. Evaluation of clinical teaching and professional development in a problem and community-based nursing module. Curationis. 2004;27(1):82-93.

12. Uys L, Gwele N. Curriculum development in nursing: Process and innovation: Routledge; 2004.

13. Mtshali G. Conceptualisation of community-based basic nursing education in South Africa: a grounded theory analysis. Curationis. 2005;28(2):5-12.

14. Feenstra C, Gordon B, Hansen D, Zandee G. Managing community and neighborhood partnerships in a community-based nursing curriculum. Journal of Professional Nursing. 2006;22(4):236-41.

15. Mtshali N. Implementing community-based education in basic nursing education programs in South Africa. Curationis. 2009;32(1):25-32.

16. Wee LE, Yeo WX, Tay CM, Lee JJ, Koh GC. The pedagogical value of a student-run community-based experiential learning project: the Yong Loo Lin School of medicine public health screening. Annals Academy of Medicine Singapore. 2010;39(9):686-91.

17. Kaye DK, Muhwezi WW, Kasozi AN, Kijjambu S, Mbalinda SN, Okullo I, et al. Lessons learnt from comprehensive evaluation of community-based education in Uganda: a proposal for an ideal model community-based education for health professional training institutions. BMC Medical Education. 2011;11:1-9.

18. Mwanika A, Okullo I, Kaye D, Muhwezi W, Atuyambe L, Nabirye R, et al. Perception and valuations of community-based education and service by alumni at Makerere university college of health sciences. BMC International Health and Human Rights. 2011;11(1):1-8.

19. Ladhani Z, Scherpbier AJ, Stevens FC. Competencies for undergraduate community-based education for the health professions–a systematic review. Journal of Medical Teacher. 2012;34(9):733-43.

20. Hamad B. Community-based Education: What? Why? How? International Handbook of Medical Education: A Guide for Students. 2012(Chapter 20):201.

21. Linda NS, Mtshali NG, Engelbrecht C. Lived experiences of a community regarding its involvement in a university community-based education programme. Curationis. 2013;36(1):1-13.

22. Nowak M, Fitzpatrick J, Schmidt C, DeRanieri J. Community partnerships: teaching volunteerism, emergency preparedness and awarding Red Cross certificates in nursing school curricula. Procedia-Social and Behavioral Sciences. 2015;174:331-7.

23. Kim MT, Kim KB, Huh B, Nguyen T, Han H-R, Bone LR, et al. The effect of a community-based self-help intervention: Korean Americans with type 2 diabetes. American Journal of Preventive Medicine. 2015;49(5):726-37.

24. Stricklin SM. Achieving clinical competencies through community-based clinical experiences. Journal of the American Psychiatric Nurses Association. 2016;22(4):291-301.

25. Amalba A, Van Mook W, Mogre V, Scherpbier A. The perceived usefulness of community based education and service (COBES) regarding students’ rural workplace choices. BMC Medical Education. 2016;16(1):1-11.

26. Mtshali NG, Gwele NS. Community-based nursing education in South Africa: A grounded-middle range theory. Journal of Nursing Education Practice. 2016;6(2):55-67.

27. Lubbers J, Rossman C. The effects of pediatric community simulation experience on the self-confidence and satisfaction of baccalaureate nursing students: A quasi-experimental study. Nurse Education Today. 2016;39:93-8.

28. Lubbers J, Rossman C. Satisfaction and self-confidence with nursing clinical simulation: Novice learners, medium-fidelity, and community settings. Nurse Education Today. 2017;48:140-4.

29. Heydari H, Rahnavard Z, Ghaffari F. Exploring the position of community-based nursing in Iran: a qualitative study. International Journal of Community Based Nursing and Midwifery. 2017;5(4):386-96.

30. Asakawa T, Kawabata H, Kisa K, Terashita T, Murakami M, Otaki J. Establishing community-based integrated care for elderly patients through interprofessional teamwork: a qualitative analysis. Journal of Multidisciplinary Healthcare. 2017;10:399-407.

31. Massimi A, De Vito C, Brufola I, Corsaro A, Marzuillo C, Migliara G, et al. Are community-based nurse-led self-management support interventions effective in chronic patients? Results of a systematic review and meta-analysis. PloS one. 2017;12(3):e0173617.

32. Cohn J, Corrigan J, Lynn J, Meier D, Miller J, Shega J, et al. Community-based models of care delivery for people with serious illness. National Academy of Medicine Perspectives. 2017:1-13.

33. Parry YK, Hill P, Horsfall S. Assessing levels of student nurse learning in community based health placement with vulnerable families: Knowledge development for future clinical practice. Nurse Education in Practice. 2018;32:14-20.

34. Huang W, Long H, Li J, Tao S, Zheng P, Tang S, et al. Delivery of public health services by community health workers (CHWs) in primary health care settings in China: a systematic review (1996–2016). Global Health Research and Policy. 2018;3(1):1-29.

35. Harvey C, Hegney D, Sobolewska A, Chamberlain D, Wood E, Wirihana L, et al. Developing a community-based nursing and midwifery career pathway–A narrative systematic review. Journal of PloS one. 2019;14(3):e0211160.

36. Walker LO, Avant KC. Strategies for theory construction in nursing. edition t, editor: Pearson; 2019.

37. Kamau M, Mirie W, Kimani S, Mugoya I. Effect of community based health education on knowledge and attitude towards iron and folic acid supplementation among pregnant women in Kiambu County, Kenya: a quasi experimental study. PloS One. 2019;14(11):e0224361.

38. Claramita M, Setiawati EP, Kristina TN, Emilia O, Van Der Vleuten C. Community-based educational design for undergraduate medical education: a grounded theory study. BMC Medical Education. 2019;19(1):1-10.

39. Steffy ML. Community health learning experiences that influence RN to BSN students interests in community/public health nursing. Public Health Nursing. 2019;36(6):863-71.

40. Higgins K, Hawkins J, Horvath E. Improving oral health: integrating oral health content in advanced practice registered nurse education. The Journal for Nurse Practitioners. 2020;16(5):394-7.

41. Lestari E, Scherpbier A, Stalmeijer R. Stimulating students’ interprofessional teamwork skills through community-based education: a mixed methods evaluation. Journal of Multidisciplinary Healthcare. 2020;13:1143-54.

42. Cheng Y-C, Huang L-C, Yang C-H, Chang H-C. Experiential learning program to strengthen self-reflection and critical thinking in freshmen nursing students during COVID-19: A quasi-experimental study. International Journal of Environmental Research Public Health. 2020;17(15):5442.

43. Aroogh MD, Shahboulaghi FM. Social participation of older adults: a concept analysis. International Journal of Community Based Nursing and Midwifery. 2020;8(1):55.

44. Shin HY, Kim KY, Kang P. Concept analysis of community health outreach. BMC Health Services Research. 2020;20(1):1-9.

45. Ohta R, Ryu Y, Sano C. The contribution of citizens to community-based medical education in Japan: A systematic review. International Journal of Environmental Research Public Health. 2021;18(4):1575.

46. Barasteh S, Rassouli M, Karimirad MR, Ebadi A. Future challenges of nursing in health system of Iran. Frontiers in Public Health. 2021;9:1052.

47. Shirvani T, Javadivala Z, Azimi S, Shaghaghi A, Fathifar Z, Devender Bhalla H, et al. Community-based educational interventions for prevention of type II diabetes: a global systematic review and meta-analysis. Systematic Reviews. 2021;10(1):1-12.

48. Kerrigan V, Kelly A, Lee A, Mungatopi V, Mitchell A, Wyber R, et al. A community-based program to reduce acute rheumatic fever and rheumatic heart disease in northern Australia. BMC Health Services Research. 2021;21(1):1-5.

49. Hall B, Tandon R. Social responsibility and community based research in higher education institutions. Socially Responsible Higher Education: Brill; 2021. p. 1-18.

50. Nagata C, Tsutsumi M, Kiyonaga A, Nogaki H. Evaluation of a training program for community-based end-of-life care of older people toward aging in place: a mixed methods study. Nurse Education in Practice. 2021;54:103091.

51. Fauziddin M, Suryanti S, Wiryanto W. Community-based education and regional culture, has it been put into practice? AL-ISHLAH: Jurnal Pendidikan. 2022;14(2):1069-78.

52. Nuuyoma V, Munangatire T, Nghiweni N. Nursing students’ experiences of community-based learning in an undergraduate programme at a Namibian university. International Journal of Africa Nursing Sciences. 2022;17:100458.

53. Ng JAI. A look back: assessment of the learning outcomes of the community-based research experiences of the senior high school students of a higher education institution in Batangas. International Journal of Learning, Teaching and Educational Research. 2022;21(3):342-58.

54. Atashzadeh-Shoorideh F, Zeydani A, Hosseini M, Zohari-Anboohi S. Explaining of existing challenges of community-based undergraduate nursing education in Iran: a qualitative study. BMC Medical Education. 2023;23(1):1-12.
